# Investigation of chromosome 1q reveals differential expression of members of the S100 family in clinical subgroups of intracranial paediatric ependymoma

**DOI:** 10.1038/sj.bjc.6604651

**Published:** 2008-09-09

**Authors:** V Rand, E Prebble, L Ridley, M Howard, W Wei, M-A Brundler, B E Fee, G J Riggins, B Coyle, R G Grundy

**Affiliations:** 1 Children's Brain Tumour Research Centre, University of Nottingham, Nottingham, NG7 2UH, UK; 2 West Midlands Regional Genetics Lab, BWH, Birmingham, B15 2TG, UK; 3 Cancer Research UK Institute for Cancer Studies, University of Birmingham, Birmingham, B15 2TT, UK; 4 Department of Pathology, Birmingham Children's Hospital, Birmingham, B4 6NH, UK; 5 Department of Medicine, Duke University Medical Center, Durham, NC 27710, USA; 6 Department of Neurosurgery, Johns Hopkins University School of Medicine, Baltimore, MD 21231, USA

**Keywords:** SAGE, CGH, differential expression, ependymoma, 1q, S100 proteins

## Abstract

Gain of 1q is one of the most common alterations in cancer and has been associated with adverse clinical behaviour in ependymoma. The aim of this study was to investigate this region to gain insight into the role of 1q genes in intracranial paediatric ependymoma. To address this issue we generated profiles of eleven ependymoma, including two relapse pairs and seven primary tumours, using comparative genome hybridisation and serial analysis of gene expression. Analysis of 656 SAGE tags mapping to 1q identified CHI3L1 and S100A10 as the most upregulated genes in the relapse pair with *de novo* 1q gain upon recurrence. Moreover, three more members of the S100 family had distinct gene expression profiles in ependymoma. Candidates (CHI3L1, S100A10, S100A4, S100A6 and S100A2) were validated using immunohistochemistry on a tissue microarray of 74 paediatric ependymoma. In necrotic cases, CHI3L1 demonstrated a distinct staining pattern in tumour cells adjacent to the areas of necrosis. S100A6 significantly correlated with supratentorial tumours (*P*<0.001) and S100A4 with patients under the age of 3 years at diagnosis (*P*=0.038). In conclusion, this study provides evidence that S100A6 and S100A4 are differentially expressed in clinically relevant subgroups, and also demonstrates a link between CHI3L1 protein expression and necrosis in intracranial paediatric ependymoma.

Identification of cancer-specific molecular alterations has had a major impact on understanding the biology of cancer and improving treatment options in many cancers. For example, therapeutic agents such as Imatinib (Gleevec®) targeting genes altered in the cancer cell, but not in normal cells, are increasingly being tested in the clinical setting. By systematically deciphering the genomes of different cancers, frequent genomic aberrations and gene expression changes have been revealed and correlated with clinical details. However, the biological and clinical relevance of most aberrations in cancer is largely unknown. One such cancer, where the investigation of the tumour-specific genetic aberrations would have a major benefit upon the understanding of the disease that could lead to better treatment choices and patient survival, is ependymoma.

Ependymoma is the third most common brain tumour of childhood, with around 50% occurring in infants younger than 5 years of age (Bouffet *et al*, 1998). Treatment and prognostication is predominantly currently based on clinical criteria despite many genomic studies identifying common molecular aberrations (Reardon *et al*, 1999; Zheng *et al*, 2000; Scheil *et al*, 2001; Ward *et al*, 2001; Carter *et al*, 2002; Dyer *et al*, 2002; Grill *et al*, 2002; Jeuken *et al*, 2002; Gilhuis *et al*, 2004; Taylor *et al*, 2005; Suarez-Merino *et al*, 2005; Mendrzyk *et al*, 2006; Modena *et al*, 2006; Sowar *et al*, 2006; Lukashova-v Zangen *et al*, 2007). Currently complete tumour resection is the only confirmed independent prognostic marker, indicating a better patient outcome (Bouffet *et al*, 1998; Sala *et al*, 1998). Despite complete resection, local recurrence is reported in up to 50% of paediatric cases (McLaughlin *et al*, 1998; van Veelen-Vincent *et al*, 2002). Some improvements in survival rates have been seen over the last 30 years, with some 50% of patients now obtaining 5-year survival (Gatta *et al*, 2003, 2005). However, when compared with other cancers such as acute lymphoblastic leukaemia, where more than 80% of children are long-term survivors, these improvements lag far behind. There is a need to identify robust biological markers and to better understand the biology of ependymoma to improve therapeutic strategies and patient survival.

Gain of 1q is one of the most common genomic aberrations in cancer (Struski *et al*, 2002) and is frequently gained in ependymoma, occurring at an incidence of >20% (Reardon *et al*, 1999; Scheil *et al*, 2001; Ward *et al*, 2001; Dyer *et al*, 2002; Grill *et al*, 2002). The gain of the whole of the q-arm of chromosome 1 has been associated with a poor prognosis in ependymoma (Dyer *et al*, 2002), and has also been shown to adversely affect patient survival in other paediatric cancers, including Wilms' tumour and Ewing's sarcoma (Hirai *et al*, 1999; Hing *et al*, 2001). The region-specific amplicon, 1q25, has been demonstrated as an independent prognostic marker, indicating a poor prognosis (Mendrzyk *et al*, 2006). However, the mechanisms by which 1q, or 1q25, confer adverse biological behaviour in ependymoma is unclear and a more detailed analysis of 1q is necessary.

Chromosome 1q gain has also been shown to be the most common global genetic change in ependymoma recurrent tumours, seen in 67% of cases, with *de novo* gain of 1q frequently occurring in the relapse sample (Dyer *et al*, 2002). The region-specific amplicon 1q21.1–q32.1 has been associated with tumour recurrence in intracranial ependymoma (Mendrzyk *et al*, 2006). Several other region-specific amplicons frequently gained in ependymoma include 1q21.3–q23.1, 1q21–q31 and 1q22–q31, 1q31.1–q31.3, 1q31–q32 and 1q41–qter (Kramer *et al*, 1998; Scheil *et al*, 2001; Ward *et al*, 2001; Mendrzyk *et al*, 2006; Modena *et al*, 2006). The 1q32 amplicon contains two genes laminin and GAC1 that are overexpressed in ependymoma and the candidate gene DUSP12 is located within the frequently gained ‘hotspot’ 1q23.3 (Suarez-Merino *et al*, 2005; Mendrzyk *et al*, 2006). Despite these observations the role of these amplicons remain unclear and, to date, no specific genes on 1q have been shown to directly relate to ependymoma tumorigenesis, relapse or patient outcome.

Taken together this evidence implicates the gain of 1q as a marker for adverse clinical behaviour. However, the underlying biology and the gene(s) involved remain to be elucidated. To address these issues we used a combination of comparative genome hybridisation (CGH) and serial analysis of gene expression (SAGE) to identify candidate genes on 1q. Candidates were validated using immunohistochemistry (IHC) on a tissue microarray and the protein expression levels correlated with clinicopathological data to determine their potential role in intracranial paediatric ependymoma.

## Materials and methods

### Sample cohort

For SAGE and CGH analysis, 11 fresh-frozen tumour samples were obtained from the Duke Brain Tumour Bank, USA and Birmingham Children's Hospital, UK (Table 1). Five normal brain libraries (white matter, cerebral cortex, paediatric frontal cortex and two cerebellum) and six other brain tumour types (one astocytoma grade I, eight astrocytoma grade II, 11 astrocytoma grade III, 10 glioblastoma, two oligodendroglioma and 20 medulloblastoma) were downloaded from the SAGE Genie website (http://cgap.nci.nih.gov/SAGE; Boon *et al*, 2002).

For immunohistochemistry, a tissue microarray (TMA) was constructed using formalin-fixed paraffin-embedded (FFPE) tumour material from 74 primary tumours. The samples were obtained from the Histopathology Department at the Birmingham Children's Hospital and further Neuropathology Departments of the Children's Cancer Leukaemia Group (CCLG) Centres. The histology of each tumour was verified, representative areas were identified by a pathologist (MAB) and a minimum of three cores were taken. Haematoxylin and Eosin smears of corresponding frozen material were used to confirm viable tumour. Clinical information was obtained from the CCLG Data Centre, West Midlands Children's Tumour Registry and case notes.

### CGH and SAGE libraries

Comparative genome hybridisation was performed as described by Dyer *et al* (2002). Serial analysis of gene expression libraries were constructed using the RNA isolated from 11 frozen tissue samples as described by Boon and Riggins (2003). SAGE2000 software (http://www.sagenet.org) was used to extract tags from the original sequence files and processed to remove duplicate ditags, linker sequences and repetitive tags. Tag counts and library information for nine SAGE libraries have been posted to CGAP's SAGE Genie website (http://cgap.nci.nih.gov/SAGE) (Boon *et al*, 2002). The complete list of genes mapping to chromosome 1q were downloaded from the Ensembl genome browser (NCBI36) and the best tag for each identified by searching the SAGE Genie website (http://cgap.nci.nih.gov/SAGE) using the HUGO gene symbol. All SAGE libraries were normalised to tags per 200 000 to enable cross-library comparison.

### SAGE analysis

The SAGE data was analysed in four ways: (1) Tags were identified in relapse pair R1 with a higher tag count in the relapsed sample (E1023) than in the corresponding primary (E628). This data was filtered to determine tags with either the same, or less, count in the relapse compared with the primary of R2. Tags were then ranked based on the difference between the relapse and the primary of pair R1. (2) The mean normalised tag counts were calculated for the 656 1q tags across 10 ependymoma SAGE libraries and five normal brain libraries to identify ependymoma-specific genes (E1023 was removed from this analysis). Results were then sorted based on the difference between the mean ependymoma and mean normal brain tag count. (3) The data from (2) was filtered to identify tags with ⩽0.5 in normal brain and >2 in ependymoma, then sorted by the highest ependymoma tag count. (4) The mean tag count for each S100 gene was calculated across the six different tumour types and fold changes between ependymoma and normal brain and ependymoma and other brain tumour types determined.

### Immunohistochemistry

Immunohistochemical study was performed using a tissue microarray of 74 FFPE tumours. Antibodies against the following antigens were used: S100A10 (monoclonal, 1 : 8000; Swant, Switzerland), S100A6 (monoclonal, 1 : 1000; Sigma-Aldrich), S100A4 (polyclonal, 1 : 4000; Dako, UK); S100A2 (monoclonal, 1 : 500; Sigma-Aldrich) and CHI3L1 (1 : 1000; YKL-40; Quidel Corporation, San Diego, CA, USA). Briefly, 5 μm sections were cut and incubated at 37°C for 16 h, de-paraffinised in xylene and hydrated through decreasing concentrations of ethanol. Antigen retrieval was performed in a pressure cooker in sodium citrate buffer (pH 6.0) for 1 min at full pressure. The TMAs were incubated with normal goat serum followed by an endogenous peroxidase block (Dako, Cambs, UK). The S100 antibodies were incubated overnight at 4°C. The Dako Chemate Envision Kit (Dako, Cambs, UK) was used to detect the target antigen according to the manufacturer's instructions. Sections were then counterstained with haematoxylin (Surgipath, Cambs, UK), dehydrated and mounted. The positive controls used were: for CHI3L, glioblastoma; S100A2, skin and kidney; for S100A4, appendix and liver; for S100A6 and S100A10 kidney. The slides were examined by light microscopy with × 10 and × 40 objectives (Olympus BX-41, UK) by two independent investigators including a pathologist (MAB). Sections were then scored as described in Figure 2.

### Statistical analysis

Statistical analyses were performed using SPSS v15.0. Univariate analysis of the association of protein expression levels with clinical variables (Table 4) was assessed by Fisher's exact test. Kaplan–Meier survival curves were constructed to investigate candidates as prognostic markers. Multivariate cox regression hazard analysis was used to identify independent prognostic markers. A *P*-value of <0.05 was considered statistically significant and all values are given in Table 4.

## Results

### Patients and CGH profiles

Genomic profiles were generated for 11 flash-frozen ependymoma (Table 1). Of the nine tumours with a CGH profile, six tumours (four paediatric and two adults) had a balanced genome (i.e., had no detectable genomic losses or gains) and three (two paediatric and one adult) had a structural genome (i.e., had few, mainly partial chromosome gains). Two paediatric relapse pairs, R1 and R2, were included in this study. Comparative genome hybridisation revealed that the recurrent sample (E1023) of relapse pair R1 had gain of 1q whereas the primary (E628) was balanced (i.e., *de novo* 1q gain). The genomes of the primary (E1p) and recurrent sample (E1r) of relapse pair R2 were both balanced.

### SAGE libraries and 1q tags

A total of 801 076 SAGE tags were generated from 11 ependymoma samples with, on average, over 26 000 unique tags per library. The complete libraries for 9 of the 11 ependymoma are available to download from the SAGE Genie website. The unique SAGE tags representing the genes on 1q were identified using the Ensembl genome browser, reducing the number of tags to be analysed to 656.

### Upregulated genes associated with 1q gain in recurrent ependymoma

From the filtered dataset of 656 1q tags, 205 were selected that had a higher tag count in the relapse of pair R1 than in the corresponding primary. To identify the genes upregulated on account of gain of 1q, filtering was done to select tags that were specifically upregulated upon relapse in the R1 pair compared with the relapse pair R2. This reduced the number of tags to 149. Once the tags were ranked based on the difference in tag count between the recurrent sample of pair R1 and the primary, CHI3L1, S100A10 and PSMB4 were revealed as the top three genes upregulated as a consequence of the gain of 1q in recurrent ependymoma (Table 2).

### Ependymoma-associated 1q transcripts

A comparison of 1q tags in 10 ependymoma with five normal brain libraries revealed S100A10 as the most upregulated gene (125.5 tags; Table 3). A second member of the S100 family, S100A6, was identified with one of the highest differences between ependymoma and normal brain of 27.1. CHI3L1 also ranked highly, with a difference of 25.7 tags. When the data was then filtered for tags meeting the criteria ⩽0.5 mean tag count in normal brain but >2 tags in ependymoma, the uncharacterised gene C1orf192 showed the highest difference in expression of 17.3 (Table 3). S100A4, a third member of the S100 family, was also one of the most upregulated genes in ependymoma with a difference in tag count of 6.6.

### S100 gene expression in ependymoma and other brain tumours

**Figure 1:**
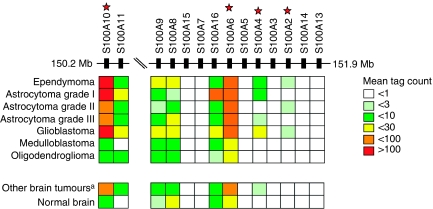
Summary of the mean SAGE tag counts for 14 S100 genes in ependymoma and six other brain tumour types (astrocytoma grade I, II, III, glioblastoma, oligodendroglioma and medulloblastoma). The S100 genes are in genomic order and the start and end positions on chromosome 1 are given in megabases (Mb). ^a^Mean of the 52 SAGE libraries from the six brain tumour types. A red star marks the genes selected for further investigation.

Our analyses revealed that several members of the same gene family were associated with 1q gain and were also upregulated in ependymoma compared with normal brain tissue. Therefore, to investigate this gene family, the mean tag counts were calculated for all 14 S100 genes located on 1q21.3 represented in SAGE genie in the ependymoma SAGE libraries (Figure 1). Of the 13 S100 genes, S100A4 had the highest mean tag count for ependymoma relative to normal brain with a fold change of 20.7 and S100A10 had the highest mean tag count of 133 in ependymoma. S100A2 was the only S100 gene that was expressed in ependymoma but not in normal brain with mean tag counts of 1.5 and zero, respectively. No expression of five members of the S100 family (A15, A7, A5, A14 and A13) was observed in ependymoma.

Members of the S100 family have been associated with different cancers, including brain tumours. Therefore, this analysis was extended to six other brain tumour types and the mean SAGE tags were calculated for the 14 S100 genes in each tumour type (Figure 1). S100A10 and S100A6 showed the highest mean expression across the six brain tumour types of 43.6 and 41.7 tags, respectively. In both grade I astrocytoma and glioblastoma (GBM) S100A10 had the highest tag count of 134 and 112.3, respectively. S100A6 had the highest tag counts in oligodendroglioma (15 tags), medulloblastoma (10.4 tags) and grade II and III astrocytoma (49.1 and 55 tags, respectively). Overall, S100A10 had the highest fold change between ependymoma and other brain tumours of three and S100A4 had the highest fold change between ependymoma and normal brain of 20.7.

### Differential expression of S100 proteins in intracranial paediatric ependymoma

**Figure 2:**
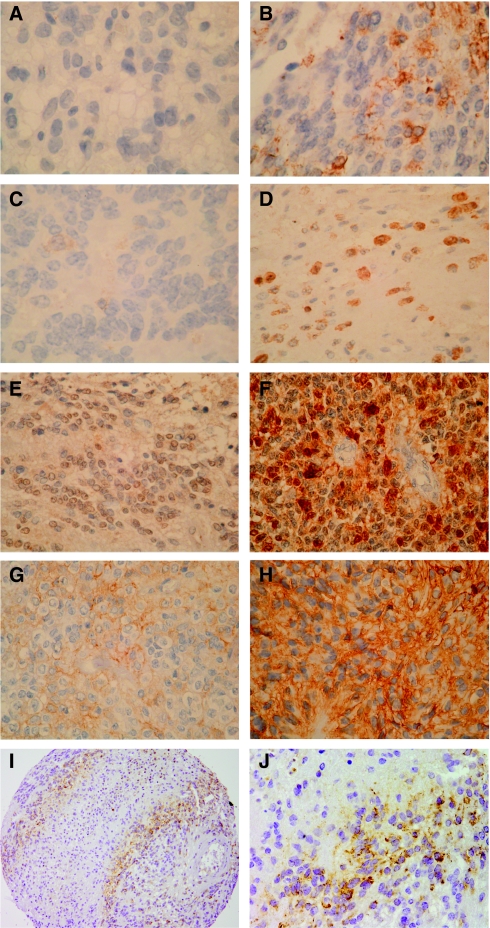
Examples of immunohistochemical staining patterns of S100A2, S100A4, S100A6, S100A10 and CHI3L1 on the paediatric ependymoma tissue microarray. The tumour-specific staining pattern for S100A2 (nuclear and/or cytoplasmic) was scored as either negative (0%; **A**) or positive (>0%; **B**). For S100A4 (nuclear and cytoplasmic staining) cores were grouped as either negative/weak (<1%; **C**) or moderate (⩾1%; **D**). S100A6 and S100A10 protein expression (cytoplasmic and/or nuclear staining for A6 and membranous staining for A10, respectively) was determined based on the percentage of immunopositive cells (0%; <10%; ⩾10% and 0%; <50%; ⩾50%, respectively) ranging in intensity from negative to strong (0 to 2+). The cumulative scores denoted the expression levels, which were grouped as either negative/weak or moderate/strong for S100A6 (**E**, **F**) and S100A10 (**G**, **H**). CHI3L1 expression (cytoplasmic, granular) was scored as either negative (0%), weak (<25%; **I**) or strong (⩾25%; **J**). Magnification × 100 for all figures except h, which is × 10.

Four members of the S100 family were selected for further investigation based on their distinct gene expression profiles in ependymoma. Protein expression levels of S100A10, S100A6, S100A4 and S100A2 were determined by immunohistochemistry using an independent cohort of seventy-four primary paediatric ependymoma arrayed on a tissue microarray (Figure 2A–H). One of the eleven ependymoma samples used to create SAGE libraries, ER1p, was represented on the TMA. In this sample, no gene or protein expression was observed for S100A2 and S100A4 with SAGE tag counts of 0 per 200 000 tags and negative protein staining observed by IHC. For S100A6 the SAGE tag count was 15 per 200 000 tags and immunostaining determined as negative/weak. Both gene and protein expression was observed for S100A10, where the SAGE tag count was 76 per 200 000 tags and moderate protein expression was observed by IHC.

Univariate analysis was performed to explore possible associations between the S100 protein expression levels and clinical variables in primary ependymoma (Table 4). S100A6 significantly correlated with tumours arising in the supratentorial region of the brain (*P*<0.001) and S100A4 correlated with age at diagnosis under 3 years (*P*=0.038). No significant correlations were found for S100A10 or S100A2. Kaplan–Meier survival analysis of the clinical parameters and S100 protein expression showed that resection status and tumour location were the only indicators of prognosis, with complete resection and supratentorial tumours indicating a better patient outcome (*P*<0.001 and *P*=0.020, respectively). Similarly, multivariate analysis revealed extent of resection and tumour location as independent prognostic markers (*P*=0.007 and *P*=0.001, respectively).

### CHI3L1 expression in ependymoma

Forty-eight primary tumours were scored for CHI3L1 protein expression. Tumours were categorised as having negative (0%), weak (<25%) or strong (⩾25%) expression levels. Twenty-eight (58%) were negative for CHI3L1 protein expression, 14 (29%) showed weak and six (13%) demonstrated strong expression. CHI3L1 protein expression was determined as weak for sample ER1p, which had a SAGE tag count of 30 per 200 000 tags. No significant correlations with the clinical parameters investigated were found. Histopathological review revealed that in five cases, where areas of necrosis were visible, CHI3L1 protein expression was restricted to the cytoplasm of viable tumour cells adjacent to the areas of necrosis (Figure 2I and J).

## Discussion

Little is known about the genes and genetic mechanisms underlying ependymoma tumorigenesis, patient relapse and survival. To address these issues we focused our study on chromosome 1q, one of the most commonly gained regions in ependymoma. Using CGH and SAGE profiling we identified CHI3L1 and members of the S100 family as candidate genes in ependymoma. Immunohistochemical analysis on a large cohort of paediatric ependymoma revealed that CHI3L1 protein expression is associated with necrosis and that members of the S100 family are differentially expressed in clinically relevant subgroups. S100A6 is significantly associated with paediatric ependymoma arising in the supratentorial compartment and S100A4 strongly correlates with patients aged less than 3 years at diagnosis.

In this study, different approaches were taken to mine the SAGE data to identify the ependymoma-associated genes on chromosome 1q. Analysis of the effect of *de novo* 1q gain on gene expression in recurrent ependymoma revealed that CHI3L1 was the most highly expressed gene. Strikingly, four members of the same gene family (S100A10, S100A6, S100A4 and S100A2) were also identified as having distinct gene expression profiles in ependymoma. Comparison of 10 ependymoma SAGE libraries with five normal brain libraries identified S100A10 as the most highly expressed gene on 1q in ependymoma; it was also the second most highly expressed gene in the relapse sample with gain of 1q. S100A6 was also identified as one of the most highly expressed genes in ependymoma when compared with ‘normal’ brain. S100A4, was implicated in ependymoma as, again, being one of the most highly expressed genes in ependymoma with very low expression in ‘normal’ brain. Analysis of all 1q S100 genes represented in SAGE Genie identified S100A2 as the only S100 gene expressed in ependymoma but not expressed at any level in ‘normal’ brain.

S100A10, S100A6, S100A4 and S100A2 are all members of the S100 family of calcium-binding proteins and are located in a cluster on 1q21.3; a region that has been shown to have both high level gains (1q21–q31; Ward *et al*, 2001) and an association with tumour recurrence in ependymoma (1q21.1–q23.1; Mendrzyk *et al*, 2006). Members of the S100 family show divergent expression patterns in a range of tissues and several have been linked with cancer, including medulloblastoma (Hernan *et al*, 2003; Lindsey *et al*, 2007) and astrocytoma (Camby *et al*, 1999, 2000). S100A6 has been shown to clearly distinguish between low (WHO grade I and II) and high (WHO grade III and IV) grade astrocytic tumours (Camby *et al*, 1999). In ependymoma, we have clearly demonstrated that S100A6 is differentially expressed in tumours arising in different locations of the brain, and is significantly associated with supratentorial tumours (*P*<0.001).

Clinically supratentorial ependymoma are associated with better survival rates when compared with posterior fossa tumours (Schiffer and Giordana, 1998). This survival difference could be because of a number of confounding factors, for example, the resectability of supratentorial when compared with infratentorial tumours (Palma *et al*, 2000). There is now evidence that ependymoma arising within different regions of the central nervous system exhibit specific and distinct genetic signatures (Taylor *et al*, 2005). For example, genes upregulated specifically in supratentorial tumours include members of the EPHB-EPHRIN and NOTCH cell-signalling systems. Our observation of the differential expression of S100A6 in different regions of the brain adds to this supratentorial gene signature.

In ependymoma we have shown that S100A4 is significantly associated with patients under the age of 3 years at diagnosis in intracranial paediatric ependymoma (*P*=0.038). Differences in the genomic profiles between tumours from patients under the age of 3 years and older children have previously been identified. For example, balanced genomes (with no detectable genomic losses and gains) are significantly associated with children younger than 3 years of age at surgery (Dyer *et al*, 2002). This finding suggests that ependymoma occurring in patients less than 3 years are biologically distinct from those occurring in older children. It has been hypothesised that tumours occurring in infants may be driven by powerful genetic events that lead to presentation at a young age without the requirement for additional genetic changes (Dyer *et al*, 2002). Although S100A4 is one of the best characterised of the S100 genes in terms of its role in cancer (Emberley *et al*, 2004; Salama *et al*, 2007), no other study has previously reported a link with patient age or explored its role in ependymoma. The significance of S100A4 in ependymomas arising in children under 3 years of age is not clear but its differential expression demonstrates that there is a distinction between genetic events occurring in children of different ages.

S100A4 and S100A6 are clearly differentially expressed in paediatric ependymoma and can be used to distinguish clinically and biologically relevant subgroups. Expanding our study to the gene expression levels across six other brain tumour types showed that for a number of S100 genes the expression levels were notably elevated in a particular tumour type. For example, S100A16 is elevated in grade I astrocytoma and S100A11 in glioblastoma. In contrast, several S100 genes had similar expression levels across multiple tumour types, including S100A14, S100A13 and S100A3. Similar expression profiles were also seen across all tumour types for S100A8 and S100A9 but this could be attributed to their function in which they form a heterodimer complex (Vogl *et al*, 2006). These findings highlight the importance of further investigation of specific members of the S100 family to understand their function in ependymoma and other brain tumours.

CHI3L1, located on 1q32.1, was the most overexpressed gene in the relapse pair with 1q gain and was also one of the most upregulated genes in ependymoma when compared with ‘normal’ brain. Immunohistochemistry of CHI3L1 revealed a correlation between the immunostaining and necrosis. Notably, the TMAs were constructed by selecting three representative areas from each tumour, in this process we tended to avoid areas of necrosis, however, in five cases necrosis was present. In these five cases staining showed that CHI3L1 was more highly expressed in the cytoplasm of tumour cells adjacent to the necrotic regions (Figure 2I and J). CHI3L1 encodes for YKL-40, which is a secreted protein that has been reported to be overexpressed in a number of different cancers, including glioma, and has been proposed as a new therapeutic target (Pelloski *et al*, 2005; Johansen *et al*, 2007). The role of CHI3L1 in cancer is unknown, but it has been suggested that it has a function in a number of pro-survival processes (Johansen *et al*, 2007). In glioblastoma (GBM), where necrosis is a characteristic, both CHI3L1 expression and necrosis are associated with poor prognosis (Burger and Green, 1987; Raza *et al*, 2002; Pelloski *et al*, 2005; Homma *et al*, 2006; Kleihues *et al*, 2007). In ependymoma, we did not find a correlation with prognosis, thus, raising the possibility that CHI3L1 is a marker of necrosis rather than of adverse biology *per se*. These observations in GBMs and our findings in ependymoma suggest a link between CHI3L1 and necrosis in brain tumours. As the cores represented on the TMA are selected to avoid regions of necrosis, our findings maybe an under-representation and further investigation of CHI3L1 expression on whole tissue sections is necessary.

Previously we identified gain of 1q as one of the most common gains in primary and recurrent ependymoma and demonstrated a tendency that patients with gain of 1q have a poorer outcome (Dyer *et al*, 2002). The aim of this study was to investigate 1q in ependymoma to gain insight into the role of genes located in this region. In this study, we have identified members of the S100 family located within the commonly gained amplicon 1q21.3 and provide evidence of their differential expression in clinical subgroups of paediatric ependymoma: S100A4 is associated with patients of a very young age at diagnosis and S100A6 with supratentorial tumour location. We also demonstrated a link between CHI3L1 protein expression and necrosis. However, we are yet to elucidate the underlying mechanism by which 1q gain confers adverse biological behaviour in paediatric ependymoma. We are now extending this study to a larger tumour cohort to further unravel the underlying biology of 1q in this complex tumour.

## Figures and Tables

**Table 1:** Summary of clinical data, SAGE libraries and CGH profiles of 11 ependymoma

					**SAGE**		**CGH**		
**Library**	**Age**	**Sex**	**P/R**	**Location**	**Total tags**	**Unique tags**	**Gains**	**Losses**	**Group**
*Relapse pair R1*									
1 E628	3	F	P	PF	120431	39836	None	None	B
2 E1023	7	F	R	PF	122690	40027	1q, 17q21-qtel	21q21-qtel	S
									
*Relapse pair R2*									
3 ER1p	2.5	M	P	PF	52490	21369	None	None	B
4 ER1r	4.7	M	R	PF	52910	20997	None	None	B
									
*Primary only*									
5 E510	1.7	F	P	PF	84073	30595	None	None	B
6 E512	7	F	P	PF	75379	27374	9, 13, 14, 17q	1	S
7 E455	17	M	P	SP	51825	19611	NS	NS	—
8 E353	27	F	P	U	73822	27211	None	None	B
9 E580	29	M	P	PF	68614	25883	NS	NS	—
10 E582	31	M	P	SP	52189	17282	None	None	B
11 E239	34	F	P	PF	46653	20459	None	6,18, 22	S

B=balanced; F=female; NS=not scoreable; M=male; P=primary; R=recurrence; PF=posterior fossa; S=structural; SP=spinal; U=unknown. SAGE libraries 3 to 11 are available to download from (http://cgap.nci.nih.gov/SAGE). Age is the age of the patient at diagnosis given in years.

**Table 2:** Most highly expressed genes in relapse pair with *de novo* 1q gain

**SAGE TAG**	**Gene ID**	**Locus**	**Gene title**	**R1p**	**R1r**	**R2p**	**R2r**	**Difference^a^**
GTATGGGCCC	CHI3L1	1q32.1	Chitinase 3-like 1 (cartilage glycoprotein-39)	38	326	30	0	288
AGCAGATCAG	S100A10	1q21.3	S100 calcium-binding protein A10	122	322	76	11	200
ATCAGTGGCT	PSMB4	1q21.3	Proteasome (prosome, macropain) subunit, *β* type, 4	41	84	41	18	43
TACTTTTGGC	SLC41A1	1q32.1	Solute carrier family 41, member 1	11	34	15	7	23
TCAGTTTGTC	HAX1	1q22	HS1-binding protein	11	34	26	18	23
GAGTGCAGGT	TROVE2	1q31.2	TROVE domain family member 2	31	53	19	18	22
TACAGCACGG	MGST3	1q24.1	Microsomal glutathione *S*-transferase 3	36	57	7	7	21
GTGTTTACGT	RAB4A	1q42.13	RAB4A, member RAS oncogene family	8	26	15	0	18
TGCAATAAGC	FAM36A	1q44	Protein FAM36A	6	24	22	15	18
TCTTTCCCCA	PHLDA3	1q32.1	Pleckstrin homology -like domain, family A, member 3	0	16	7	3	16

Columns R1p, R1r, R2p and R2r show the tags per 200 000 for the primary sample of relapse pair R1, relapse samples of relapse pair R1, primary sample of relapse pair R2 and the relapse samples of relapse pair R2, respectively.

a The difference was calculated using the formula (R1r-R1p).

**Table 3:** Most highly expressed genes associated with ependymoma

**SAGE TAG**	**Gene ID**	**Locus**	**Gene title**	**Mean tumour^a^**	**Mean normal^b^**	**Difference^c^**
*(b) Upregulated in ependymoma*						
AGCAGATCAG	S100A10	1q21.3	S100 calcium-binding protein A10	132.9	7.4	125.5
GTCTGGGGCT	TAGLN2	1q23.2	Transgelin 2	126.4	20	106.4
GGCTAATTAT	ATP1A2	1q23.2	ATPase, Na+/K+ transporting, *α* 2 (+) polypeptide	146.3	70	76.3
TTTTTAATGT	H3F3A	1q42.12	H3 histone, family 3A	96.6	24.6	72
GGCTGTACCC	CSRP1	1q32.1	Cysteine and glycine-rich protein 1	146.4	79.2	67.2
TGAAGAGAAG	PRDX6	1q25.1	Peroxiredoxin 6	47.2	6	41.2
CCCCCTGGAT	S100A6	1q21.3	S100 calcium-binding protein A6 (calcyclin)	38.3	11.2	27.1
GTATGGGCCC	CHI3L1	1q32.1	Chitinase 3-like 1 (cartilage glycoprotein-39)	26.5	0.8	25.7
TACATTCTGT	MCL1	1q21.2	Myeloid cell leukaemia sequence 1 (BCL2-related)	27.4	5	22.4
TAATTCTTCT	CCT3	1q23.1	Chaperonin containing TCP1, subunit 3 (*γ*)	40.4	20.8	19.6
						
*(c) Upregulated in ependymoma with ⩽0.5 mean tag count in normal brain*						
ATCCAGACAG	C1orf192	1q23.3	Chromosome 1 open reading frame 192	17.3	0	17.3
CCCAGATGAT	SLC39A1	1q21.3	Solute carrier family 39 (zinc transporter), member 1	8.5	0.4	8.1
ATTCCTTTTT	FMO3	1q24.3	Flavin containing monooxygenase 3	7.2	0	7.2
TAAGTCTATA	FCGR2A	1q23.3	Fc fragment of IgG, low affinity IIa, receptor for (CD32)	6.6	0	6.6
TTCAAGATAC	ANGPTL1	1q25.2	Angiopoietin-like 1	6.6	0	6.6
ATGTGTAACG	S100A4	1q21.3	S100 calcium-binding protein A4	5.9	0.4	5.5
AGTGGTGGCT	FMOD	1q32.1	Fibromodulin	5.7	0.4	5.3
ATCACACAGC	LMOD1	1q32.1	Leiomodin 1 (smooth muscle)	4.4	0	4.4
TAGAAGGTGG	C1orf54	1q21.2	Chromosome 1 open reading frame 54	4.8	0.4	4.4
GAAGCCAATG	DISP1	1q41	Dispatched homologue 1 (Drosophila)	3.5	0.4	3.1

All tag counts are normalised per 200 000 tags.

a Mean tag count per individual tumour SAGE library.

b Mean tag count per normal brain SAGE library.

c Difference between mean tag count per ependymoma library and normal brain libraries.

**Table 4:** Patient demographics, univariate and multivariate analysis of clinical and biological factors of 74 primary paediatric intracranial ependymoma

**S100 protein**			**Gender**		**Tumour location**		**WHO grade**		**Age<3 years at diagnosis^a^**		**Resection status**	
**>Patient demographics**	**Sc**	**%**	**M**	**F**	**ST**	**PF**	**II**	**III**	**<3**	**>3**	**C**	**IC**
No.	—	—	41	33	19	55	49	35	34	40	26	44
%	—	—	55.4	44.6	25.7	74.3	66.2	33.8	45.9	54.1	37.1	62.9
**>Univariate analysis**			***P*** **-value**		***P*** **-value**		***P*** **-value**		***P*** **-value**			
*S100A2*			1.000		1.000		1.000		0.551		—	
Positive	14	21										
Negative	51	79										
Total scored	65	—										
												
*S100A4*			0.598		0.252		0.788		**0.038**		—	
Negative/weak	41	64										
Moderate	23	36										
Total scored	64	—										
												
*S100A6*			0.549		**0.000**		1.000		0.236		—	
Negative/weak	55	81										
Moderate/strong	13	19										
Total scored	68	—										
												
*S100A10*			0.589		0.12		0.783		0.788		—	
Negative/weak	21	35										
Moderate/strong	39	65										
Total scored	60	—										
												
>*Multivariate analysis*												
Hazard ratio	—	—	0.905		0.219		1.01		1.124		5.357	
95% CI	—	—	0.451–1.816		0.072–0.662		0.448–2.278		0.561–2.251		2.080–13.792	
*P*-value	—	—	0.778		**0.007**		0.981		0.742		**0.001**	

C=complete resection; CI=confidence interval; F=female; IC=incomplete resection; M=male; No.=number; PF=posterior fossa; Sc=samples scored; ST=supratentorial.

a The median age at diagnosis of the primary tumours was 3.8 years (range, 8 months to 14.9 years). Significant *P*-values (<0.05) are given in bold.
